# Expression of T-cell immunoreceptor with immunoglobulin and tyrosine-based inhibitory motif domains (TIGIT) in anaplastic thyroid carcinoma

**DOI:** 10.1186/s12902-022-01113-4

**Published:** 2022-08-15

**Authors:** Tadao Nakazawa, Takuya Nagasaka, Keita Yoshida, Atsuko Hasegawa, Feng Guo, Di Wu, Kenzo Hiroshima, Ryohei Katoh

**Affiliations:** 1 Department of Pathology, Tokyo Women’s Medical University Yachiyo Medical Center (TYMC), 477-96 Owada-Shinden, Yachiyo-shi, Chiba, 276-8524 Japan; 2 Department of Pathology, Ito Hospital, Shibuya, Tokyo 150-8308 Japan

**Keywords:** TIGIT, Anaplastic thyroid carcinoma, Poorly differentiated thyroid carcinoma, Medullary thyroid carcinoma, Immunohistochemistry

## Abstract

**Background:**

Immune checkpoint proteins have not been fully examined in follicular cell-derived thyroid carcinoma and medullary thyroid carcinoma (MTC). Anaplastic thyroid carcinoma (ATC) is one of the most aggressive carcinomas. Even multimodal treatment does not result in favorable clinical outcomes for patients with ATC. Anti-tumor immunity has therefore been highlighted as having therapeutic promise for ATC.

**Methods:**

We examined a novel immune checkpoint receptor, T-cell immunoreceptor with immunoglobulin and tyrosine-based inhibitory motif domains (TIGIT), in variable thyroid lesions: adenomatous goiter, follicular adenoma, and thyroid carcinoma (TC) using immunohistochemistry (IHC).

**Results:**

Our IHC results showed that TIGIT expression was detected in cancer cells of MTC and high-grade TC: poorly differentiated thyroid carcinoma (PDTC) and ATC. Neoplastic cells were positive for TIGIT in four of five MTCs (80.0%), 17 of 31 ATCs (54.8%) and in 3 of 12 PDTCs (25.0%). TIGIT was not detected in any adenomatous goiters, thyroid benign tumors, or differentiated thyroid carcinoma (DTCs). Intriguingly, ATC cells showing pleomorphic/giant cell features were positive for TIGIT, while ATC cells with other cell morphologies lacked the immunoreactivity. Intra-tumoral immune cell was inclined to be enriched in TIGI-positive ATC. Although coexisting papillary thyroid carcinoma (PTC) components demonstrated high-grade microscopic features, neither the PTC nor follicular thyroid carcinoma (FTC) components expressed TIGT in any composite ATCs.

**Conclusion:**

TIGIT was immunohistochemically found in MTC with high frequency and partially in high-grade TC. TIGIT expression in cancer cells may be beneficial for a potential utility in MTC and a subset of high-grade TC, especially ATC therapy.

## Introduction

Anaplastic thyroid carcinomas (ATCs) account for up to 2% of thyroid carcinomas (TCs) and are highly malignant epithelial tumors that result in poor patient clinical outcomes [1]. Sufficient efficacy has not been obtained despite multimodal therapeutic strategies for ATC [2]. Differentiated thyroid carcinoma (DTC) includes papillary thyroid carcinoma (PTC) and follicular thyroid carcinoma (FTC), and comprises almost all thyroid cancers. In contrast to ATC, the prognosis of patient with DTC is greatly favorable. Poorly differentiated thyroid carcinoma (PDTC) is morphologically and behaviorally intermediate between DTC and ATC [3]. Medullary thyroid carcinoma (MTC) is a rare C-cell derived neuroendocrine tumor producing calcitonin. It is clinically diagnosed at more advanced stage and behaves more aggressively than DTC [4]. Generally, ATC and PDTC are recognized as high-risk thyroid carcinomas (TCs).

Extremely poor prognosis necessitates novel therapeutic approaches for ATC. Anti-tumor immune checkpoint proteins, such as PD-1/PDL1 and CTLA-4, are targets for cancer immunotherapy and have been examined in various malignant tumors. Twelve patients with progressive ATC were treated with kinase inhibitor plus pembrolizumab [5]. Salvage pembrolizumab therapy improved progression free survival (PFS) and overall survival (OS). Clinical trial using spartalizumab, a humanized monoclonal antibody against PD-1, started as part of a phase I/II cohort study in patients with advanced/metastatic solid tumor [6]. Nineteen percent of the patients responded to the therapy. The response rate was significantly higher in patients with PD-L1-positive ATC than negative, regardless of *BRAF* mutation status in ATC. More recently, Hatashima et al*.* confirmed clinical efficacy of pembrolizumab or nivolumab in a subset of patients with locally advanced and metastatic unresectable ATC [7].

T-cell immunoreceptor with immunoglobulin and tyrosine-based inhibitory motif domains (TIGIT) has recently been recognized as a novel immune checkpoint receptor [8]. It binds to CD155, its ligand, and is a co-inhibitory transmembrane glycoprotein belonging to poliovirus receptor (PVR)/-nectin superfamily. TIGIT negatively regulates effector T cell activity and leads to immune tolerance for tumor cell proliferation and expansion. It has been shown to be expressed on active T cells and natural killer cells (NK cells), including tumor cells [9].

ATC generally occurs via multiple steps as consequence of the accumulation of variable genetic and epigenetic alterations. This highly aggressive carcinoma has a wide variety of characteristic microscopic features [10]. An inflammatory background is common in ATC compared to other histological subtypes of TCs. Heavy infiltration of macrophages is usually encountered in ATC, and may facilitate tumor progression [11, 12]. Based upon these genetic and morphological characteristics, an ATC-specific immune microenvironmental milieu may contribute to immune escape from cytotoxic activity and facilitate ACT cell growth.

In TCs, the representative immune checkpoint proteins, PD-1/PD-L1, have been intensively investigated in numerous previous reports [2, 13–15]. According to these previous reports, ATCs highly express PD-1/PD-L1, implying that these molecules are candidate therapeutic targets. Giannini et al*.* reported that expression of inhibitory immune checkpoint mediators including TIGIT was significantly high in ATCs at mRNA level [16]. Nonetheless, little is known about other immune checkpoint proteins in TCs. Therefore, we examined TIGIT expression in TCs with immunohistochemistry (IHC) using formalin-fixed paraffin-embedded tissues.

## Methods

### Human thyroid tissues

We examined a total of 105 surgically resected specimens and/or biopsies for thyroid nodules. The specimens consisted of seven adenomatous goiters (AGs), 13 follicular thyroid adenomas (FTAs), five medullary thyroid carcinomas (MTCs), 11 follicular thyroid carcinomas (FTCs), 28 papillary thyroid carcinomas (PTCs), 12 PDTCs, and 31 ATCs. All PDTC, ATC, and MTC samples were obtained from patients who underwent surgery or biopsy at Ito Hospital. The other thyroid tissues were surgical material from Tokyo Women’s Medical University Yachiyo Medical Center (TYMC). Tumor stages were adopted from the *TMN Classification of Malignant Tumours, 8th Edition* [17].

All excised materials and biopsies were routinely processed and embedded in paraffin blocks. Sections were cut 4 µm thick and then stained with hematoxylin and eosin for routine pathological diagnosis. After microscopic observation, we selected paraffin blocks that included the maximum cut surface of thyroid nodules for immunohistochemistry. Each pathological diagnosis was made based upon histopathological findings and immunohistochemical results according to the *World Health Organization classification of tumours of endocrine organs* [3] and recent review [10]. In particular, diagnoses of PDTC were established stringently depending upon the Turin proposal of 2007 [18]. Immunohistochemical examinations, including PAX8, were performed and all results of PDTCs were consistent with those previously reported [19]. All samples were evaluated independently by experienced pathologists (T. N. and R. K.).

### Immunohistochemistry

Four-micrometer-thick sections were cut from the paraffin blocks, deparaffinized, and then pretreated with EDTA-buffer at pH 9.0 for 20 min. The sections were incubated with rabbit monoclonal antibody against TIGIT (catalog no. ab243903, Abcam, Cambridge, UK) according to the manufacturer’s instruction. Simultaneously, some ATC tissues were incubated with pancytokeratin AE1/AE3 (Dako, Carpinteria, CA) on their corresponding serial sections. We used 3,3’-diaminobenzidine tetrahydrochloride (Sigma, St Louis, MO, USA) for specific immunostaining, and finally counterstained with hematoxylin. The total staining process was carried out on a Leica BOND-MAX immunostainer (Leica Biosystems, Newcastle, UK). Tonsil tissue was used as a positive control, and an additional section from the corresponding block without primary antibody was used as a negative control for TIGIT. The same two pathologists also reviewed the positive and negative controls.

### Evaluation of immunohistochemistry

We considered cells with membranous and/or cytoplasmic immunoreactivity for TIGIT to be positive, and semi-quantitatively analyzed each case by determining the percentage of positive epithelial cells. Each case was categorized into three groups: 0) less than 1%, 1 +) 1–49%, and 2 +) more than 50%.

### Statistical analysis

We used χ^2^ tests to compare the frequencies at which differentiated TCs (PTCs and FTCs) and ATCs had TIGIT-positive cancer cells. The frequencies of differentiated TCs and high-grade TCs (PDTCs and ATCs) were also compared using the same method (Table 1). Significance was set at *P* < 0.05. Data analysis was performed with SPSS version 11.0 for Windows (SPSS, Tokyo, Japan).

**Table 1:** Summary of TIGIT expression in epithelial component of adenomatous goiters and thyroid tumors

histological subtype	number of case (*n*)	positive cases (%)
adenomatous goiter	7	0
follicular adenoma	13	0
medullary carcinoma	5	4 (80.0%)
follicular carcinoma	11	0^*†^
papillary carcinoma	26	0^*†^
poorly differentiated carinoma	12	3 (25.0%)†
anaplastic carcinoma	31	17 (54.8%)*†

^*^P < 0.05 (papillary carcinoma and follicular carcinoma versus anaplastic carcinoma)

^†^*P* < 0.05 (papillary carcinoma and follicular carcinoma versus poorly differentiated carcinoma and anaplastic carcinoma)

## Results

### TIGIT in non-neoplastic thyroid tissues

**Fig. 1:**
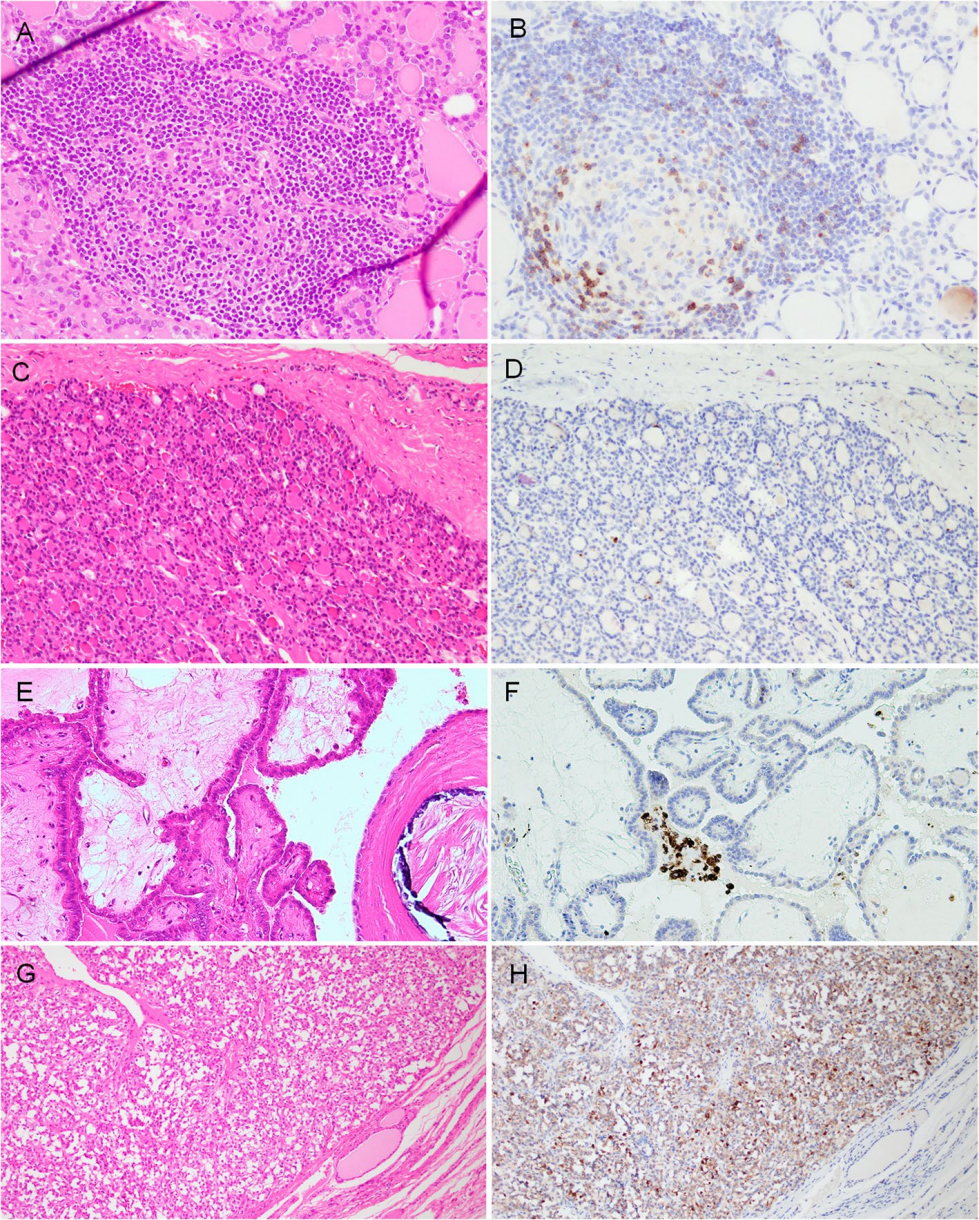
Representative microphotographs of TIGIT immunostaining in non-neoplastic and neoplastic thyroid tissues. Non-neoplastic thyroid tissue (H&E staining) (**A**). Macrophages were diffusely positive for TIGIT. TIGIT-positive small lymphocytes were scattered in the non-neoplastic thyroid tissue (**B**). Follicular thyroid adenoma (**H**&**E**) (**C**). The tumor cells lacked immunoreactivity for TIGIT (**D**). Papillary thyroid carcinoma (**H**&**E**) (**E**). These neoplastic cells were negative for TIGIT, while the macrophages were positive (**F**). Medullary thyroid carcinoma (**H**&**E**) (**G**). Tumor cells were diffusely positive for TIGIT (**H**)

Macrophages were strongly and diffusely positive in lymphoid follicles of non-neoplastic thyroid tissue. Small-sized lymphocytes were sparsely positive. Non-neoplastic follicular cells including adenomatous goiter completely lacked immunoreactivity for TIGIT (Figs. 1A and 1B).

### TIGIT in the epithelial component of thyroid neoplasms

**Fig. 2:**
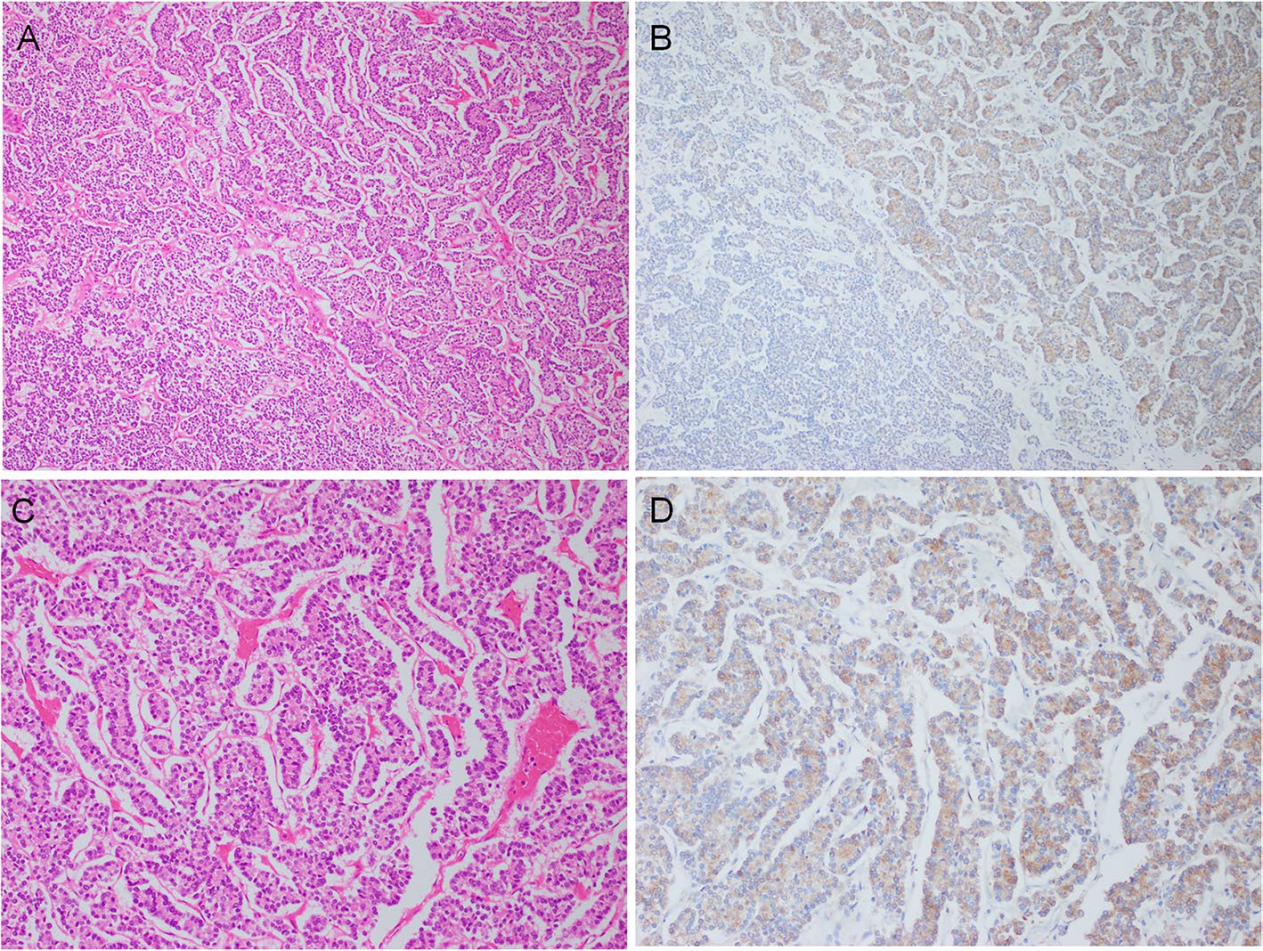
Representative immunohistochemical results of TIGIT in poorly differentiated thyroid carcinoma (PDTC). Neoplastic cells were arranged in a solid or trabecular architecture (**A**) (H&E, low magnification). About half of PDTC cells were positive for TIGIT in this area (upper and right) (**B**). TIGIT-positive area of PDTC (H&E, high magnification) (**C**). Cytoplasmic TIGIT reactivity was observed (**D**)

**Fig. 3:**
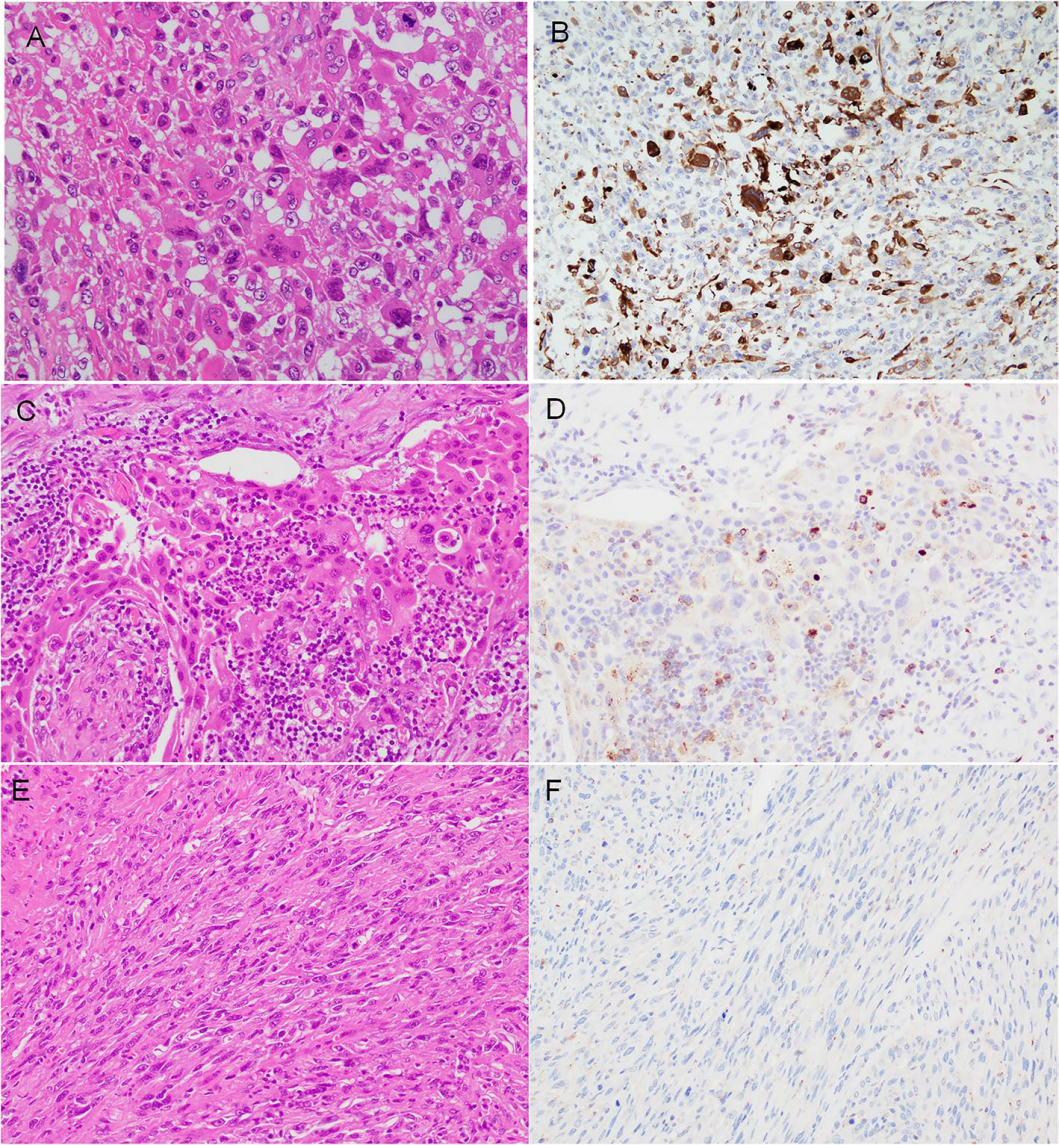
Representative results of TIGIT immunostaining in anaplastic thyroid carcinoma (ATC). Pleomorphic/giant cell-shaped tumor cells (H&E staining) (**A**). ATC cells were stained with strong intensity (**B**). Epithelioid/squamoid tumor cells (**H**&**E**) (**C**). The tumor cells were negative for TIGIT, while scattered lymphocytes were positive (**D**). Neoplastic cells displayed a spindle-shaped, sarcomatoid feature (**H**&**E**) (**E**). These ATC cells uniformly lacked immunoreactivity for TIGIT (**F**)

**Fig. 4:**
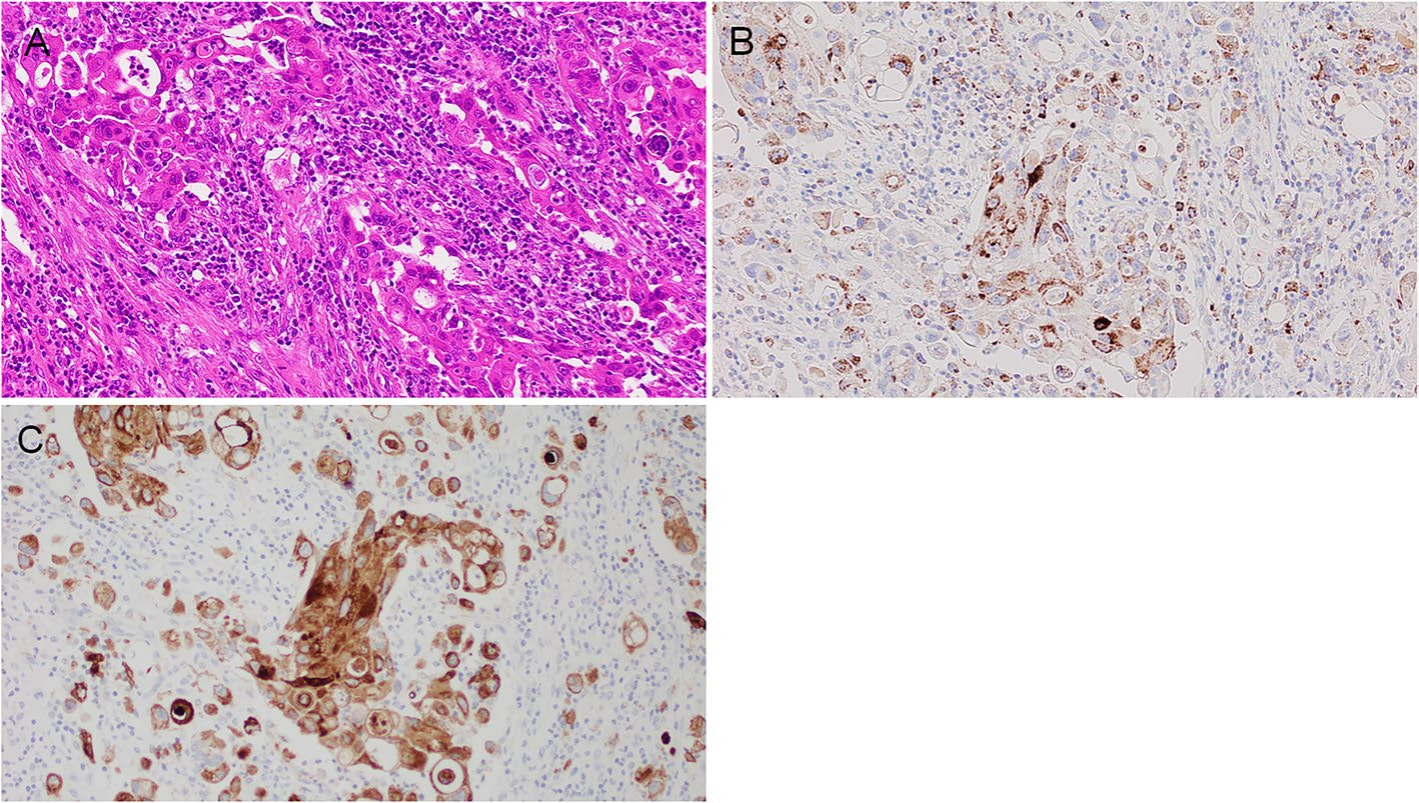
Microphotographs of ATC on H&E- (**A**), TIGIT- (**B**), and AE1/AE3- (**C**) stained serial sections. ATC cells harbored pleomorphic large nuclei and abundant eosinophilic cytoplasm with a heavy infiltrate of immune cells (**A**). Tumor cells were concurrently positive for TIGIT (**B**) and pancytokeratin AE1/AE3 (**D**)

**Fig. 5:**
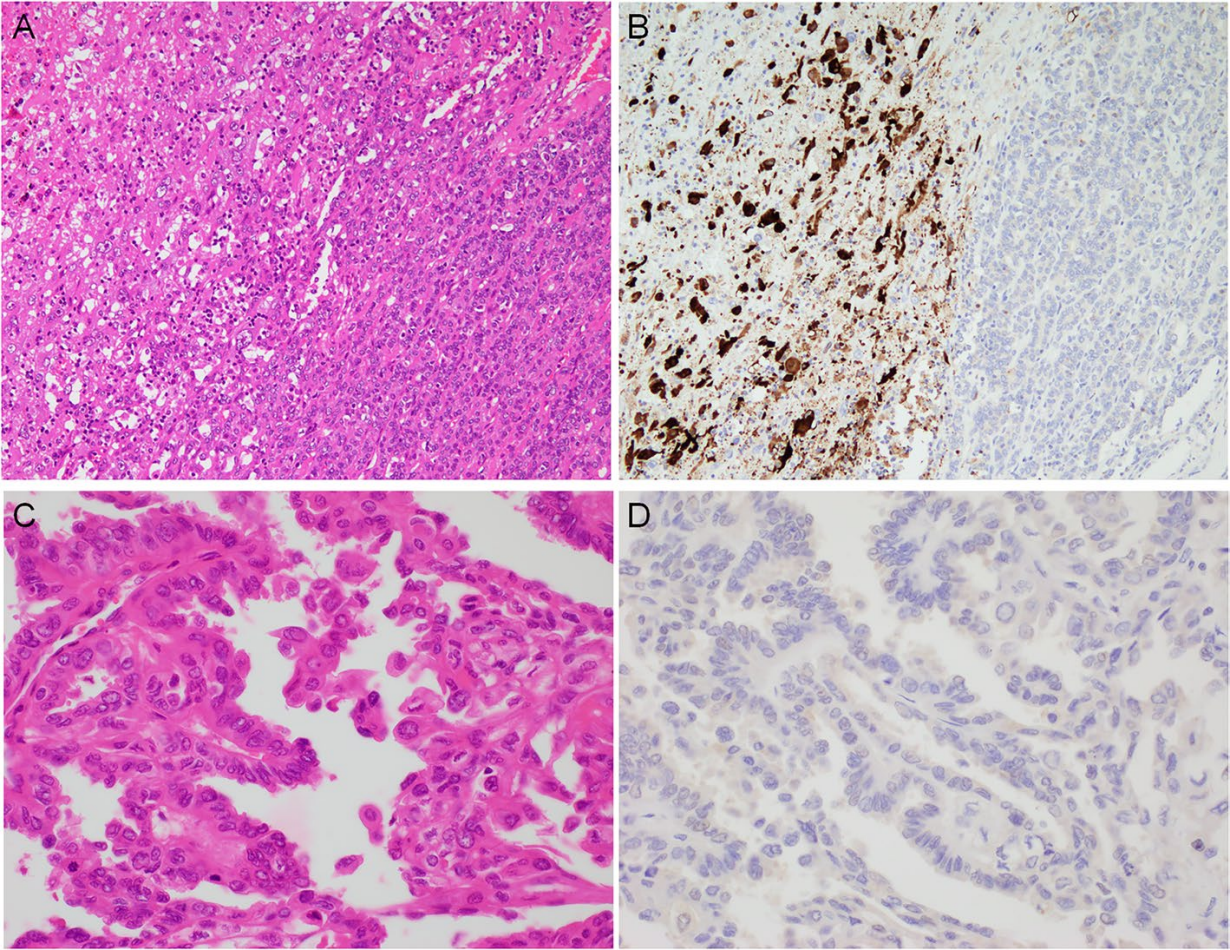
TIGIT immunohistochemical results of composite type ATCs. In H&E-stained sections, ATC components showing pleomorphic/giant cell shape (left) were concomitantly seen with adjacent FTC components (right) (**A**). The FTC cells were completely negative for TIGIT, while ATC cells were starkly positive (**B**). In PTC-composite ATC, the PTC cells showed tall cell morphology with partial hobnail structures (H&E) (**C**). These neoplastic cells completely lacked immunoreactivity for TIGIT (**D**)

TIGIT expression in the epithelial component of variable thyroid lesions is summarized in Table 1. No epithelial expression was detected in AG, FTA (Figs. 1C and 1D) and DTCs: FTC and PTC (Figs. 1E and 1F). C cell-derived neoplastic cells were positive in four of five MTCs (Table 1, Figs. 1G and 1H). Three of 12 (25.0%) PDTCs were focally positive (Tables 1 and 2, Fig. 2). ATC cells were positive in 17 of 31 cases (54.8%) (Tables 1 and 3, Figs. 3–5). Epithelial expression of TIGIT in ATC was confirmed by pancytokeratin AE/AE3 staining in serial sections (Fig. 4). The proportion of the TIGIT-positive TC was significantly higher in ATCs and high-grade TCs (PDTC and ATC) than in DTCs (*P* < 0.05) (Table 1).

**Table 2:** Summary of TIGIT expresion in neoplastic cells of 12 poorly differentiated thyroid carcinomas (PDTCs)

case no	positive tumor cell morphology	TMN stage	immune cell			IHC score
			type of infiltrate	density	predominant cell type	
1	round nuclei	T4aN0M1	scattered	low	lymphcyte	1 +
2	large and convoluted nuclei	T3NXM0	focal	low	lymphcyte	1 +
3	round neclei	T3NXM0	scattered	low	lymphcyte	1 +
4	round nuclei	T3N1bM0	scattered	low	lymphcyte	0
5	round or oval nuclei	T3N1bM1	scattered	low	lymphcyte	0
6	round or oval nuclei	T3NXM0	scattered	low	lymphcyte	0
7	round or oval nuclei	T2NXN0	scattered	low	lymphcyte	0
8	round nuclei	T3NXM0	scattered	low	lymphcyte	0
9	round or oval nuclei	T3N1aM0	scattered	low	lymphcyte	0
10	round and oval nuclei	T3NXM0	scattered	low	lymphcyte	0
11	large and convoluted nuclei	T3N1aM0	scattered	low	lymphcyte	0
12	round or oval nuclei	T3N0M0	focal	high	lymphcyte	0

**Table 3:** Summary of TIGIT expression in 34 anaplastic thyroid carcinomas (ATCs)

case no	predominat tumor cell morpology	TMN stage	positive tumor cell morphology	immune cell			IHC score
				type of infiltrate	density	predominat cell	
1	pleomorphic/giant	T4bN1bM1	pleomorphic/gaint	diffuse	high	macrophage	2 +
2	pleomorphic/giant	T4bN0MO	pleomorphic/gaint	diffuse	high	lymphocyte	2 +
3	composite (FTC)	T4bN1bM0	pleomorphic/gaint	diffuse	high	lymphocyte	2 +
4	pleomorphic/giant	T4aN0M0	pleomorphic/gaint	diffuse	high	neutrophil	2 +
5	pleomorphic/giant	T4bN0MO	pleomorphic/gaint	diffuse	high	lymphocyte	1 +
6	epithelioid/squamoid	T4bN1aM1	pleomorphic/gaint	diffuse	high	lymphocyte	1 +
7	spindle	T4bN1bM0	pleomorphic/gaint	diffuse	low	lymphocyte	1 +
8	composite (PTC)	T4bN1M1	pleomorphic/gaint	diffuse	low	lymphocyte	1 +
9	pleomorphic/giant	T4aN1bM0	pleomorphic/gaint	diffuse	low	lymphocyte	1 +
10	pleomorphic/giant	T4aNXMX	pleomorphic/gaint	diffuse	high	neutrophil	1 +
11	epithelioid/squamoid	T4aN1M0	pleomorphic/gaint	diffuse	high	lymphocyte	1 +
12	spindle	T4aN1M0	pleomorphic/gaint	diffuse	high	lymphocyte	1 +
13	spindle	T4aN1bM0	pleomorphic/gaint	diffuse	high	lymphocyte	1 +
14	spindle	T4aNXMX	pleomorphic/gaint	diffuse	high	lymphocyte	1 +
15	composite (FTC)	T3bNXM0	pleomorphic/gaint	diffuse	high	macrophage	1 +
16	pleomorphic/giant	TXNXM0	pleomorphic/gaint	focal	low	lymphocyte	1 +
17	spindle	N/A	pleomorphic/gaint	diffuse	low	lymphocyte	1 +
18	pleomorphic/giant	T4bN1bM1		diffuse	low	neutrophil	0
19	epithelioid/squamoid	T4bN1M1		diffuse	high	lymphocyte	0
20	pleomorphic/giant	T4aN1aM0		diffuse	low	macrophage	0
21	spindle	T4aN1bM1		scattered	low	lymphocyte	0
22	epithelioid/squamoid	T2N1aM1		diffuse	high	lymphocyte	0
23	spindle	T1aNXM1		diffuse	high	lymphocyte	0
24	composite (PTC)	TXN1bM0		diffuse	low	lymphocyte	0
25	composite (PTC)	TXNXM1		diffuse	high	macrophage	0
26	composite (FTC)	N/A		diffuse	high	lymphocyte	0
27	epithelioid/squamoid	N/A		diffuse	low	lymphocyte	0
28	epithelioid/squamoid	N/A		focal	low	lymphocyte	0
29	epithelioid/squamoid	N/A		diffuse	low	lymphocyte	0
30	epithelioid/squamoid	N/A		diffuse	high	lymphocyte	0
31	others (rhabdoid)	N/A		diffuse	low	macrophage	0

N/A: Not available

### TIGIT expression in cancer cell of PDTC

Table 2 summarizes TIGIT expression in the epithelial component of PDTCs. The PTCD cells were arranged in a solid/trabecular/insular (STI) pattern. The neoplastic cells uniformly present as round or oval in most PDTCs (Fig. 2). TIGIT-positive PDTC cells occupied less than half of the whole tumor in all positive PDTCs. All positive PDTCs had IHC scores of 1 + . In regards with profile of intra-tumoral immune cell and tumor stage, there was no significant difference between TIGIT-positive group and -negative.

### Microscopic features of ATC cell

Examined ATCs microscopically displayed a wide variety of tumor cell morphology, and each cell type was concomitant to a variable degree (Table 3). Based upon the morphological features of neoplastic cell, we briefly categorized five types: 1) spindle, 2) pleomorphic/giant, 3) epithelioid/squamoid, 4) composite, and 5) others. Spindle cell, pleomorphic/giant, and epithelioid/squamoid types constituted 7, 9, and 8 of 31 ATCs, respectively. A DTC component (3 PTC and 3 FTC) coexisted in 6 ATCs. These ATCs were thus classified as "composite" type. One ATC preferentially had a rhabdoid shape and was categorized into "others".

### TIGIT expression in ATC

A summary of epithelial TIGIT expression in 31 ATCs is shown in Table 3. The expression clearly depended upon cell morphology (Fig. 3, Table 3). Pleomorphic/giant cell-shaped ATC cells showed immunoreactivity in 17 of 31 ATCs in various proportions (Figs. 3A and 3B, Table 3). These cells had IHC scores of 1 + and 2 + in 13 and four ATCs. Epithelioid/squamoid ATC cells were prone to show a nonspecific reactivity due to abundant eosinophilic cytoplasm and were interpreted to be negative for TIGIT (Figs. 3C and 3D). Spindle-shaped, sarcomatoid ATC cells were completely negative for TIGIT (Figs. 3E and 3F).

Intra-tumoral immune cells, such as lymphocyte, macrophage, and neutrophil, were diffuse and dense in 12 of 17 TIGIT-positive ATCs (70.6%) in contrast to six of 14 TIGIT-negative (42.9%). Regarding tumor stage, 14 of 15 TIGIT-positive ATCs (93.3%) were operated at pT4 stage, while four of six -negative (66.7%) in available cases.

### TIGIT expression in composite ATCs

In six composite cases, the DTC component (three FTCs and three PTCs) was included in the same immunohistochemically stained sections. TIGIT expression was not detected in any FTC component of composite ATCs (Figs. 5A and 5B). TIGIT was also negative for three PTC components, although the components were high-grade PTC: tall cell variants with hobnail features (Figs. 5C and 5D). In summary, all composite DTC areas failed to display immunoreactivity for TIGIT.

## Discussion

In this study, we examined the TIGIT expression in TCs via immunohistochemistry. Our findings showed that the expression in tumor cells was detected in most MTC, about half of ATCs, and PDTCs with lower frequency despite being negative in benign lesions/tumors and DTCs. Of note, tumor cell morphology seemed to determine TIGIT expression in ATC.

Recently, TIGIT expression has been intensively examined in malignant tumors of many organs: skin melanoma [20], Hodgkin’s lymphoma [21], hepatocellular carcinoma [22], glioblastoma [23], and gastric cancer [24]. According to these previous reports, its expression is elevated in accordance with advanced tumor stage, tumor aggressiveness, poor tumor differentiation, and high propensity for lymph node metastasis. It is also adversely correlated with patient clinical outcomes. Like these carcinomas, TIGIT mRNA expression was upregulated in ATCs [16]. Our findings confirmed the evidence by immunohistochemistry. Blockade of TIGIT with a monoclonal antibody increases antitumoral effector T cells and delays tumor growth in vitro [25]. Thus, TIGIT-targeted therapy may also suppress ATC progression and proliferation and improve the survival of ATC patients. Interestingly, our IHC revealed that four of five MTCs expressed TIGIT, while low frequency (3.0%) of MTC reported in a large series by IHC [26]. According to the report, the same monoclonal antibody against TIGIT was used for immunostaining, but combined positive score (CPS) was adopted for the evaluation of IHC results. All MTCs analyzed in this study were composed of dense neoplastic and scant immune cells and, we counted only TIGIT-positive neoplastic cells. Therefore, the results might be underestimated in the report.

Most previous reports documenting TIGIT expression have evaluated immune cells, not tumor cells [20–24]. In our study, high TIGIT expression was tended to be observed in ATCs with diffuse and dense infiltrate of immune cell and advanced TMN stage. Therefore, effector T cell may exert in TIGIT-high ATCs, although functional exhaustion of immune cells is difficult to estimate merely from morphological aspects. Several reports have found that TIGIT functionally impairs glucose metabolism in CD8 effector T cells and subsequently induces their exhaustion and inhibits their antitumor effect [24, 25]. TIGIT inhibition promotes effector T cell survival via activation of the AKT/mTOR pathway. Cytokine production is also increased by TIGIT blockade [24]. Another previous report provides evidence that TIGIT deprives NK cell function through ZAP70 and ERK1/2 [27]. In addition, ATC showed an immunosuppressed microenvironment with exhausted immune cells and decreased cytokine production [16]. An effective anti-tumor immune response may also be provoked in ATC by metabolic impairment of intra-tumoral TIGIT-positive immune cells.

Regarding TIGIT in neoplastic cells, Sun et al. reported that lung adenocarcinoma cell highly expressed TIGIT by Western blot analysis using cell lines in accordance with unfavorable clinical outcomes [9]. Several researchers have reported that tumor cell expressed other immune checkpoint receptors. Non-small-cell lung cancer cells and uveal melanoma cells expressed PD-1 [28, 29]. More recently, PD-1 expression in cancer cells have been verified in ATC [30]. In addition, cell lines from various malignant tumors generate CTLA-4, which promotes apoptosis via interaction with its ligand [31]. TIGIT is similar to CTLA-4, since it participates in a complex involving other immune checkpoint receptors (CD96/TACTILE and CD112R/PVRIG) and a competing costimulatory receptor (CD226/DNAM-1) sharing multiple ligands such as CD155 and CD122 [8]. Blockade of PD-1/PDL1 and CTLA-4 in neoplastic cells clinically has satisfactory clinical effectiveness. Therefore, it may be beneficial to develop inhibitory treatments that target TIGIT, which is expressed in MTC, PDTC, and ATC cells.

The immune system plays a role in regulating tumor initiation and progression in cancer [32]. In this study, TIGIT expression was detected in high-risk TCs (PDTC and ATC), while DTC cells were thoroughly negative. In particular, ATC cells more frequently displayed TIGIT immunoreactivity than PDTC. Interestingly, DTC components (PTC and FTC) were devoid of TIGIT in composite ATC carcinomas, although the composite PTC components displayed high-grade features, tall cell with hobnail structure. Interestingly, PDTCs infrequently expressed TIGIT. Therefore, it can raise possibility that TIGIT expression increases according to aggressiveness of follicular cell-derived thyroid cancer. TIGIT immunoreactivity may have a diagnostic value in discriminating higher risk TC including MTC from DTC.

In regard to other immune checkpoint proteins, ATC shows high PD-1 expression on tumor-infiltrating immune cells, and tumor cells highly express its ligand, PD-L1 [12]. PD-L1 expression is apparently higher in ATC than in DTC at the protein and mRNA levels [13, 14]. High PD-L1 in tumor cells and low PD-1 in immune cells are significantly related to worse prognosis [12]. Moreover, PD-1 blockade in ATC cell inhibits tumor growth via the intrinsic SHP2/Ras/MAPK signaling pathway [29]. Since the PD-1/PD-L1 pathway seems to be concurrently activated in ATC, a combination of TIGIT and PD-1/PD-L1 blockade may exert synergistic anti-tumor effects on ATC cell.

## Conclusions

Immunohistochemical TIGIT expression was detected in neoplastic cells of MTC with high prevalence, about half of ATC, a quarter of PDTC. It is necessary to examine other checkpoints that constitute the complex regulatory network of TIGIT in MTC and high-grade TC, especially ATC. 

## Data Availability

The datasets　are available from the corresponding author on reasonable request.

## Abbreviations

TIGIT: T-cell immunoreceptor with immunoglobulin and tyrosine-based inhibitory motif domains
ATC: Anaplastic thyroid carcinoma
IHC: Immunohistochemistry
PDTC: Poorly differentiated thyroid carcinoma
DTC: Differentiated thyroid carcinoma
PTC: Papillary thyroid carcinoma
MTC: Medullary thyroid carcinoma
FTC: Follicular thyroid carcinoma
TC: Thyroid carcinoma
PD-1: Programmed death receptor-1
PD-L1: Programmed death receptor-ligand 1
CTLA-4: Cytotoxic T-lymphocyte antigen-4
